# Clinico-biological-radiomics (CBR) based machine learning for improving the diagnostic accuracy of FDG-PET false-positive lymph nodes in lung cancer

**DOI:** 10.1186/s40001-023-01497-6

**Published:** 2023-12-02

**Authors:** Caiyue Ren, Fuquan Zhang, Jiangang Zhang, Shaoli Song, Yun Sun, Jingyi Cheng

**Affiliations:** 1 Department of Nuclear Medicine, Shanghai Proton and Heavy Ion Center, Shanghai, 201315 China; 2 Shanghai Key Laboratory of Radiation Oncology (20dz2261000), Shanghai, China; 3 Shanghai Engineering Research Center of Proton and Heavy Ion Radiation Therapy, Shanghai, China; 4 Department of Nuclear Medicine, Shanghai Proton and Heavy Ion Center, Fudan University Cancer Hospital, Shanghai, 201315 China; 5 Center for Biomedical Imaging, Fudan University, Shanghai, China; 6 Shanghai Engineering Research Center of Molecular Imaging Probes, Shanghai, China

**Keywords:** Clinico-biological-radiomics, Machine learning, [^18^F]FDG-PET/CT, False positive rate, Mediastinal–hilar lymph nodes

## Abstract

**Background:**

The main problem of positron emission tomography/computed tomography (PET/CT) for lymph node (LN) staging is the high false positive rate (FPR). Thus, we aimed to explore a clinico-biological-radiomics (CBR) model via machine learning (ML) to reduce FPR and improve the accuracy for predicting the hypermetabolic mediastinal–hilar LNs status in lung cancer than conventional PET/CT.

**Methods:**

A total of 260 lung cancer patients with hypermetabolic mediastinal–hilar LNs (SUVmax ≥ 2.5) were retrospectively reviewed. Patients were treated with surgery with systematic LN resection and pathologically divided into the LN negative (LN-) and positive (LN +) groups, and randomly assigned into the training (*n* = 182) and test (*n* = 78) sets. Preoperative CBR dataset containing 1738 multi-scale features was constructed for all patients. Prediction models for hypermetabolic LNs status were developed using the features selected by the supervised ML algorithms, and evaluated using the classical diagnostic indicators. Then, a nomogram was developed based on the model with the highest area under the curve (AUC) and the lowest FPR, and validated by the calibration plots.

**Results:**

In total, 109 LN− and 151 LN + patients were enrolled in this study. 6 independent prediction models were developed to differentiate LN− from LN + patients using the selected features from clinico-biological-image dataset, radiomics dataset, and their combined CBR dataset, respectively. The DeLong test showed that the CBR Model containing all-scale features held the highest predictive efficiency and the lowest FPR among all of established models (*p* < 0.05) in both the training and test sets (AUCs of 0.90 and 0.89, FPRs of 12.82% and 6.45%, respectively) (*p* < 0.05). The quantitative nomogram based on CBR Model was validated to have a good consistency with actual observations.

**Conclusion:**

This study presents an integrated CBR nomogram that can further reduce the FPR and improve the accuracy of hypermetabolic mediastinal–hilar LNs evaluation than conventional PET/CT in lung cancer, thereby greatly reducing the risk of overestimation and assisting for precision treatment.

**Supplementary Information:**

The online version contains supplementary material available at 10.1186/s40001-023-01497-6.

## Background

Lobectomy with mediastinal systematic lymph node dissection (SND) is standard surgical strategy for lung cancer [1, 2]. Nevertheless, the significance of SND is controversial. The American College of Surgeons Oncology Group Z0030 trial revealed that there was no survival difference between patients with non-small cell lung cancer (NSCLC) who had SND or systematic sampling, with the 5-year disease-free survival rates were 68% and 69%, respectively (*p* > 0.05) [3]. Ishiguro et al*.* [4] and Ray et al*.* [5] also reported the similar findings: SND did not provide additional survival benefit. Central to avoid “overtreatment” (i.e., unnecessary SND) and provide a more precise and individualized lymph node (LN) dissection strategy is an accurate evaluation of node status at the mediastinal and hilar levels, especially the negative status [6].

The negative predictive value (NPV) of invasive endoscopic techniques is still unsatisfactory currently due to the difficulty of the selection suspected LN caused by the anatomical complexity of mediastinum, the location and size of LN, and the poor repeatability, etc. [7, 8]. Non-invasive imaging techniques, including chest computed tomography (CT) and [^18^F]-fluorodeoxyglucose (FDG) positron emission tomography (PET)/CT, are commonly used for LN staging [9]. PET/CT evidently has significantly higher accuracy than CT, especially with the superior NPV greater than 85% [10]. However, the main problem of PET/CT evaluation is the false-positive (FP) findings caused by non-specific FDG uptake in non-neoplastic processes such as granulomas or other inflammatory diseases, especially when intrapulmonary lesions and mediastinal–hilar LNs are both FDG-positive, with the false positive rate (FPR) of 19 ~ 22% [11, 12]. The resulting overestimation of FP LNs would have a major impact on the patient’s further treatment strategy, including unnecessary resection of benign nodules and inappropriate exclusion of surgical treatment. Thus, FP FDG studies for LN staging are inevitable.

Previous radiomics analyses based on PET/CT have demonstrated the great potential of assessing the lymph node metastasis (LNM) in lung cancer using the machine learning (ML) algorithms to exhaust the full underlying information of non-invasive medical images [13–15]. However, only a few radiomics researches have focused on the evaluation of hypermetabolic mediastinal–hilar LNs status [16]. The results in our previous study also indicated the feasibility of PET/CT radiomics in achieving “pathology-like” diagnosis non-invasively in lung cancer. Furthermore, we found that clinico-biological-radiomics (CBR) data could evaluate the tumor heterogeneity more comprehensively due to the combination of multi-scale characteristics of tumors [17]. Multi-scale and high-dimensional features need appropriate filter strategies to reduce redundancy while ensuring model effectiveness [18]. On the basis of successfully screening features and establishing excellent models using the least absolute shrinkage and selection operator (Lasso) algorithm in our previous study, we added the minimum-redundancy maximum-relevance (mRMR) algorithm before Lasso to initially narrow the range of redundant and irrelevant features in this study, which contributed to the robustness of research.

Thus, the purpose of this study was to seek a more reliable, scalable and non-invasive biomarker-based CBR data via ML algorithms to reduce the FPR and improve the accuracy for predicting the hypermetabolic mediastinal–hilar LNs status in lung cancer.

## Methods

### Patients

This retrospective study reviewed the charts of 1280 patients with single pulmonary nodule examined by [^18^F]FDG-PET/CT scanning less than 30 days before curative surgery between January 2018 and December 2022, and finally identified 260 patients of resectable T1–4 lung cancer with complete baseline clinico-biological information. The retrospective study was approved by the Ethics Committee of Shanghai Proton and Heavy Ion Center, and informed consent was waived.

**Fig. 1:**
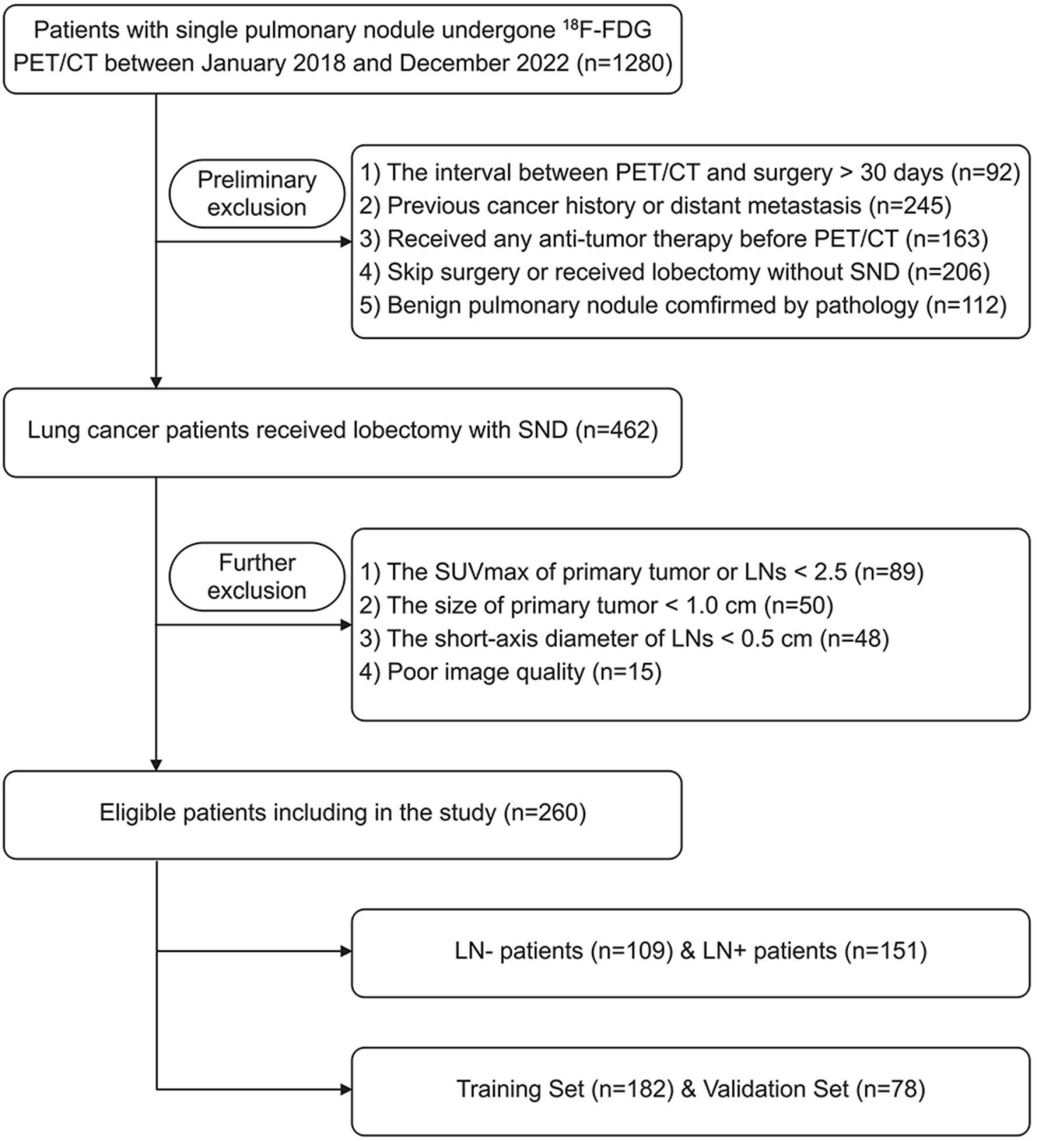
Flowchart showing the patient selection and exclusion

The specific inclusion criteria were as follows: (1) both the single intrapulmonary lesion and mediastinal–hilar LNs were FDG-positive with the maximum standardized uptake value (SUVmax) ≥ 2.5 [19, 20], the size of lesion > 1.0cm while the short axis diameter of target LNs > 0.5cm to ensure the quality of image and radiomics data; (2) first pathologically diagnosed of a primary lung cancer [21]; (3) postoperative pathological (*p*) N staging determined by SND as pN0-2 (N0: no regional LN involvement, N1: ipsilateral peribronchial, interlobar, or hilar LN involvement, N2: ipsilateral mediastinal LN involvement) [22]. The exclusion criteria included the following: (1) anti-tumor therapy before PET/CT examination or surgery; (2) lobectomy without SND; (3) distant metastasis; (4) poor image quality. The patient recruitment process is presented in Fig. 1.

Finally, totally 260 consecutive lung cancer patients were enrolled in this study, comprising 205 males and 55 females (mean age, 62.15 ± 8.62 years, range, 27–81 years), as summarized in Table 1. Among these included patients, the most common histologic subtype was adenocarcinoma (*n* = 145, 55.77%), followed by squamous cell carcinoma (*n* = 96, 36.92%). Rarer cases of small cell lung cancer (*n* = 11, 4.23%), large cell carcinoma (*n* = 6, 2.31%) and sarcomatoid carcinoma (*n* = 2, 0.77%) were reported. The patients were pathologically divided into the LN negative (LN−, pN0, *n* = 109) and positive (LN + , pN1-2, *n* = 151) groups, and assigned to a training (*n* = 182) and test (*n* = 78) sets by the random split-sample (7:3) method. Baseline clinico-biological data of each patient were reviewed and recorded.

**Table 1:** Clinical and demographic characteristics of lung cancer patients

Characteristics	Total (*n* = 260)	LN− (*n* = 109)	LN + (*n* = 151)
Gender			
Male	205 (78.85)	85 (77.98)	120 (79.47)
Female	55 (21.15)	24 (22.02)	31 (20.53)
Age (mean ± SD, y)	62.15 ± 8.62	64.94 ± 6.98	60.10 ± 9.12
Tumor histological type			
ADC	145 (55.77)	56 (51.37)	89 (58.94)
SCC	96 (36.92)	48 (44.04)	48 (31.79)
Others	19 (7.31)	5 (4.59)	14 (9.27)
T stage			
T1	126 (48.46)	66 (60.55)	60 (39.73)
T2	83 (31.92)	26 (23.85)	57 (37.75)
T3	34 (13.08)	13 (11.93)	21 (13.91)
T4	17 (6.54)	4 (3.67)	13 (8.61)
N stage			
N0	109 (41.92)	109 (100)	0
N1	53 (20.38)	0	53 (35.10)
N2	98 (37.69)	0	98 (64.90)

Data in parentheses are percentages unless otherwise noted. *LN*−  lymph node negative, *LN*+  lymph node positive, *ADC* adenocarcinoma, *SCC*  squamous cell carcinoma, *SD*  standard deviation, *T*  tumor, *N*  node

### ***[***^***18***^***F]FDG-PET/CT image protocol***

All included patients with a blood glucose levels < 8.7 mmol/L fasted for at least 6 h before the [^18^F]FDG-PET/CT scan. The scanning protocols of this retrospective study conducted in the single center were consistent with our previous study [17], and complied with the standard clinical scanning protocols [23]. The details of image acquisition process are given in Additional file 1 and “Methods” section.

### Tumor segmentation and analysis

The target lesions in this study were hypermetabolic single primary tumor and mediastinal–hilar LNs. The volume of interest (VOI) of each primary tumor was segmented by two separated experienced nuclear medicine physicians on the PET images using the gradient-based semi-automatic contouring algorithm, named “PET_Edge”, on the Medical Image Merge software (MIM, version 6.5.4, https://www.mimsoftware.com) without knowing the pathology determined by consensus. PET_Edge has been confirmed to be the most accurate and consistent method for tumor segmentation than manual and constant threshold methods [24, 25]. Then, six metabolic parameters including minimum SUV (SUVmin), SUVmax, SUVmean, metabolic tumor volume (MTV), and total lesion glycolysis (TLG) were automatically measured from each VOI.

The highest SUVmax of LNs was also recorded for each patient, simultaneously, the size of the LN with the highest SUVmax was measured with the nodal enlargement criterion of greater than 1.0 cm in short axis diameter on a transverse CT image of the fused PET/CT [10].

### Quantitative radiomics feature extraction

Subsequently, a total of 1702 quantitative radiomics features for each VOI were automatically extracted and calculated from the PET (*n* = 851) and CT (*n* = 851) images using the “PyRadiomics” module [26], respectively. The radiomic features were divided into four groups: (1) shape (*n* = 14); (2) intensity (*n* = 18); (3) texture (*n* = 75, 24 Gy level co-occurrence matrix (GLCM), 14 Gy level dependence matrix (GLDM), 16 Gy level run length matrix (GLRLM), 16 Gy level size zone matrix (GLSZM), and 5 neighboring gray tone difference matrix (NGTDM)); and (4) wavelet-based (*W*) features obtained from the filters (*H*: high pass filter, *L*: low pass filter) applied in the *x*, *y*, *z* directions (*n* = 744). The feature extraction and its definition were in accordance with the Imaging Biomarker Standardization Initiative [27], and its details are described in Additional file 1: Table S1.

### Features dimension reduction and selection

So far, we have constructed a CBR dataset containing 1738 multi-scale features (25 clinico-biological features, 11 conventional image features, and 1702 radiomics features) for all included patients. The processes of features dimension reduction and selection were performed using the classical supervised ML algorithms in the training set. Firstly, the features with intra- and inter-class correlation coefficients (ICC) < 0.8 were excluded due to the poor consistency and reproducibility. Then, we performed the mRMR algorithm to preliminarily narrow the range of redundant and irrelevant features, and selected the top 50 features. Finally, the Lasso algorithm with tenfold cross-validation was applied to further screen the optimal features for prediction model development.

### Prediction models and individualized nomogram development and evaluation

The models for predicting the hypermetabolic mediastinal–hilar LNs status in lung cancer were developed by the multivariable regression with the Akaike’s information criterion (AIC), with prediction scores (pre-scores) of each model calculated for each patient by the linear fusion of the selected non-zero features weighted by their coefficients. The performance and clinical utility of these models were evaluated and compared by the receiver-operator characteristic curve (ROC) analysis, DeLong test, and decision curve analysis (DCA) in both the training and test sets. The area under the curve (AUC) with 95% confidence interval (CI), sensitivity, specificity, accuracy, positive predictive value (PPV), negative predictive value (NPV), FPR, and false negative rate (FNR) were calculated for each model.

For models with similar overall AUC and accuracy, a lower FPR is more clinically relevant for this study. Thus, we developed an individualized nomogram to visually quantify the risk of hypermetabolic mediastinal–hilar LNs metastasis on the basis of prediction model corresponded to this rule. Calibration curves were plotted to assess the agreement between the actual probability and predicted probability of the nomogram by bootstrapping (1000 bootstrap resamples) in both the training and test sets.

### Statistical analysis

All data analysis in this study was performed on the R software (version 4.2, http://www.r-project.org). The following packages “mRMRe”, “glmnet”, “pROC” and “rmda” were applied for mRMR, Lasso, ROC, and DCA analyses, respectively. The “rms” package was used to construct nomogram and calibration curves. Numerical data with normal distribution were expressed as mean ± standard deviation (SD) and compared using an independent *t-*test, while one with non-normal distribution was expressed as median (interquartile range) and compared using a Mann–Whitney *U* test. Categorical data were described as counts and their percentages, and compared using Fisher’s exact test or *χ*^2^ test. A two-sided *p* value < 0.05 was considered statistical significance. The study process was systematically evaluated using the radiomics quality score (RQS, range − 8 to + 36 points, https://www.radiomics.world/rqs) [28].

## Results

The quality of this study was good with the RQS of 20 (55.56%) (Additional file 1), which was better than the average of PET/CT radiomics-based lung cancer researches, all of which scored below 50% [29].

### Clinico-biological and conventional image characteristics of patients

In total, 260 lung cancer patients with both the hypermetabolic primary tumor and mediastinal–hilar LNs were eventually enrolled in this study, including 109 LN− and 151 LN + patients. The patients’ statistically significant clinico-biological-image (CBI) features in the training set are presented in Table 2, while the comparison results of a total of 36 CBI features between LN- and LN + patients in the total, training, and test sets are provided in Additional file 1: Table S2.

**Table 2:** Statistically significant clinico-biological-image of lung cancer patients

Characteristics	Training set (*n* = 182)		*p*	Test set (*n* = 78)		*p*
	LN− (*n* = 78)	LN + (*n* = 104)		LN− (*n* = 31)	LN + (*n* = 47)	
Age (mean ± SD, years)	65.10 ± 7.19^†^	60.46 ± 8.88^†^	** < 0.01**	64.52 ± 6.53^†^	59.30 ± 9.66^†^	**0.01**
Weight (kg)	63.12 ± 10.65^†^	66.92 ± 10.17^†^	**0.02**	61.45 ± 11.89^†^	66.55 ± 10.69^†^	0.05
CA153 (U/mL)	12.01 (7.85, 15.77)^‡^	13.82 (10.38, 19.81)^‡^	**0.01**	13.08 (12.02, 15.81)^‡^	16.94 (11.62, 17.46)^‡^	0.09
CEA status			**0.01**			0.25
Negative	59 (75.64)	59 (56.73)		18 (58.06)	21 (44.68)	
Positive	19 (24.36)	45 (43.27)		13 (41.94)	26 (55.32)	
LN enlarged			** < 0.01**			** < 0.01**
Negative	56 (71.79)	30 (28.85)		26 (83.87)	20 (42.55)	
Positive	22 (28.21)	74 (71.15)		5 (16.13)	27 (57.45)	
LN SUVmax	4.15 ± 1.67^†^	7.86 ± 4.11^†^	** < 0.01**	4.34 ± 1.55^†^	7.19 ± 3.96^†^	** < 0.01**

Data in parentheses are percentages unless otherwise noted. *LN*−  lymph node negative, *LN*+   lymph node positive, *SD* standard deviation, *CA*  carbohydrate antigen, *CEA*  carcinoembryonic antigen, *SUV*  standardized uptake value

^†^Values refer to mean ± standard deviation

^‡^Values refer to median (interquartile range). *P* values were the results of univariate analysis and the bold ones indicated statistical significance

LN− patients were more likely to be elderly ones with lighter body weight, while LN + patients were more likely to be younger ones with higher body weight (*p* < 0.05). Simultaneously, LN + patients generally had higher level of carbohydrate antigen (CA) 153 and higher positive rate of carcinoembryonic antigen (CEA) (cut-off value: 5.2 ng/ml) than LN− patients (*p* < 0.05). The SUVmax and size of LN were significantly related to the LN status in both the training and test sets (*p* < 0.05). There were no significant differences in other clinical characteristics (such as gender and smoking status), biological factors (such as other conventional lung cancer tumor markers levels and status, and tumor histological types), and PET/CT image features (such as the size, location and all metabolic parameters of primary tumor) between the LN− and LN + patients according to the univariate analysis (*p* > 0.05).

### Features selection and prediction models development

**Fig. 2:**
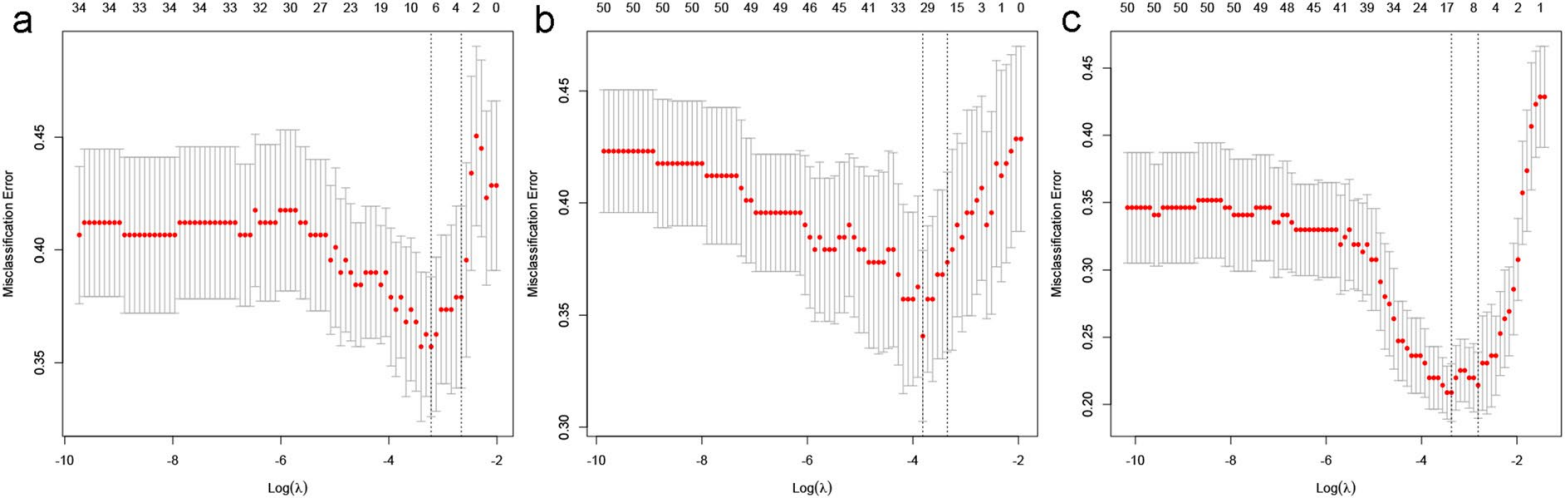
Features selection for prediction models using Lasso algorithm using tenfold cross-validation in the training set. The *X*-axis showed log (*λ*), and the *Y*-axis showed the model misclassification rate. The dotted vertical lines were drawn at the optimal values using the minimum criteria and the 1-se criteria, respectively. The 4, 19, and 7 features with non-zero coefficients were initially indicated for CBI Model (**a**), Rad Model (**b**), and CBR (**c**), respectively, according to the 1-se criteria

Two independent prediction models have been established based on the SUVmax and size of hypermetabolic mediastinal–hilar LNs, respectively. Their combination was considered as the diagnostic efficacy of PET/CT. The CBI Model was developed via 4 valuable clinical and biological features selected only using Lasso algorithm due to the low dimensionality of CBI sub-dataset with 34 features (*n* = 25 + 11–2) (Fig. 2a). Subsequently, 19 PET/CT radiomics features were selected from the radiomics sub-dataset (*n* = 1702) by the ICC rule, mRMR and Lasso algorithms sequentially in the training set (Fig. 2b), and then 10 radiomics features were confirmed by the multivariable regression with the AIC to establish the Radiomics (Rad) Model. Similarly, the CBR Model was built using the most valuable 7 clinical, biological, image, and radiomics features for predicting the hypermetabolic mediastinal–hilar LNs status in the training set (Fig. 2c). The Pre-scores of each model for each patient were calculated using the following formulas:

Pre-score (LN SUVmax) = − 2.79 + 0.57*LN SUVmax.

Pre-score (LN Enlarged) =  − 0.62 + 1.84*LN Enlarged (Negative: 0, Positive: 1).

Pre-score (LN_PET/CT) =  − 2.68 + 0.50* LN SUVmax + 0.56* LN Enlarged.

Pre-score (CBI Model) = 1.70–0.07*Age + 0.03*Weight (Kg) + 0.04*CA153 (U/mL) + 0.65*CEA status (Negative: 0, Positive: 1).

Pre-score (Rad Model) =  − 381.80 + 4.71e−09*PET_WLLH_GLCM_Cluster Shade + 312.10*PET_WHLL_GLRLM_Short Run Emphasis + 5.31* PET_WHLH_GLDM_Large Dependence Low Gray Level Emphasis − 2.23e–10*PET_WHHL_GLCM_Cluster Prominence + 71.66*PET_WHHL_GLCM_Informational Measure of Correlation 2 (Imc2) − 4.26* CT_shape_Surface Volume Ratio (SVR) + 0.03*CT _first order_90 Percentile − 0.04*CT_GLDM_Large Dependence Low Gray Level Emphasis + 0.33*CT_WLHH_first order_Median − 3.00*CT_WHHL_GLCM_MCC.

Pre-score (CBR Model) =  − 88.38 + 0.57*LN SUVmax + 0.57*LN Enlarged − 0.12*Age + 0.91*CEA status + 90.95*PET_WHHL_GLCM_Imc2 − 2.06* CT_ shape_SVR + 0.38*CT_WLLH_GLDM_Dependence Entropy.

**Fig. 3:**
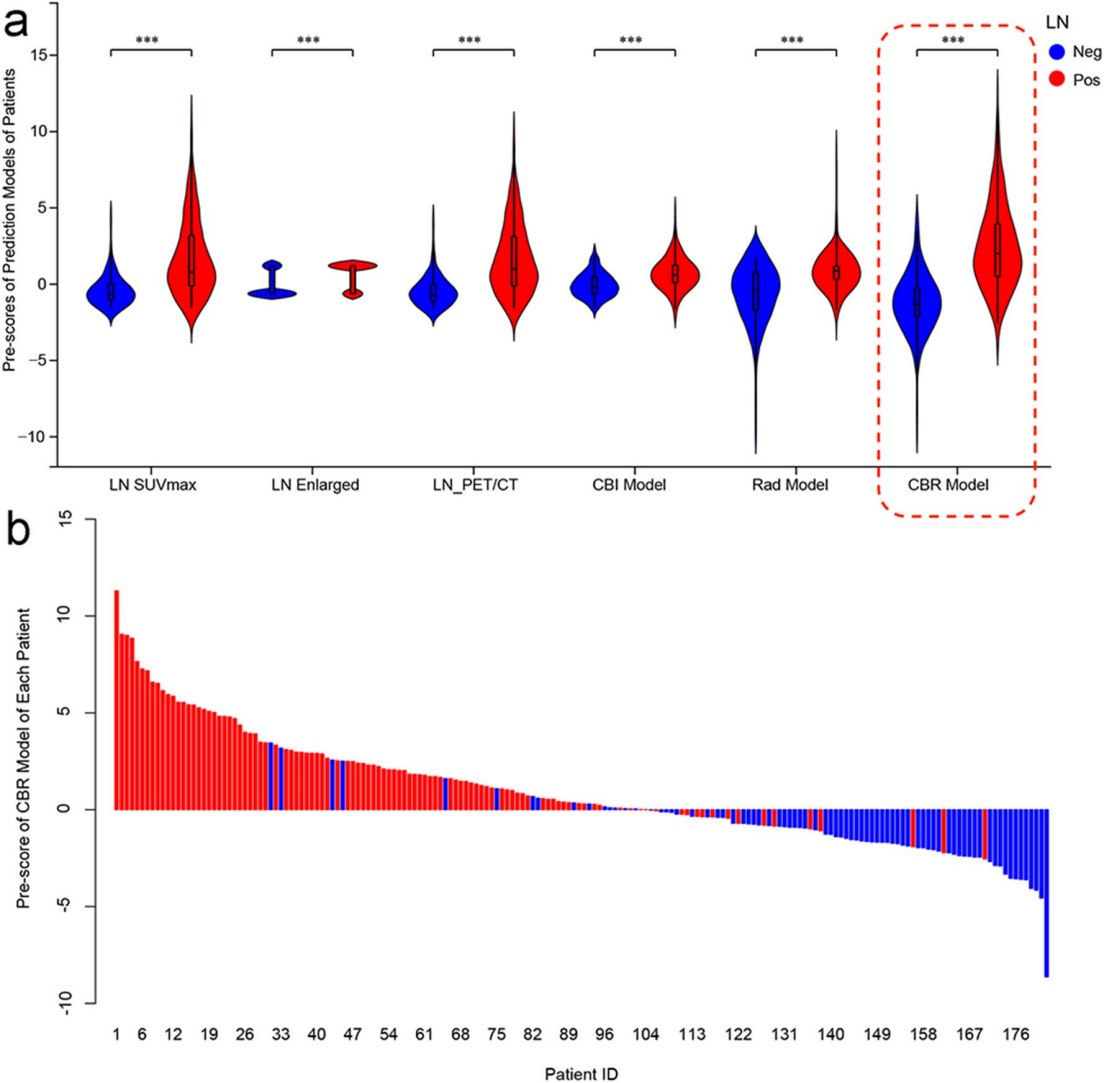
Violin plot of 6 prediction models for LN− (blue) and LN + (red) patients in training set (**a**). The black line running up and down through the violin diagram represented the range from the smallest non-outlier value to the largest non-outlier value. The waterfall plot of the CBR Model was used to visualize the distribution of the Pre-scores of individual LN− and LN + patients (**b**)

LN + patients generally had higher Pre-scores in all prediction models than those in LN− patients (*p* < 0.05, Fig. 3).

### Prediction models evaluation and comparison

**Fig. 4:**
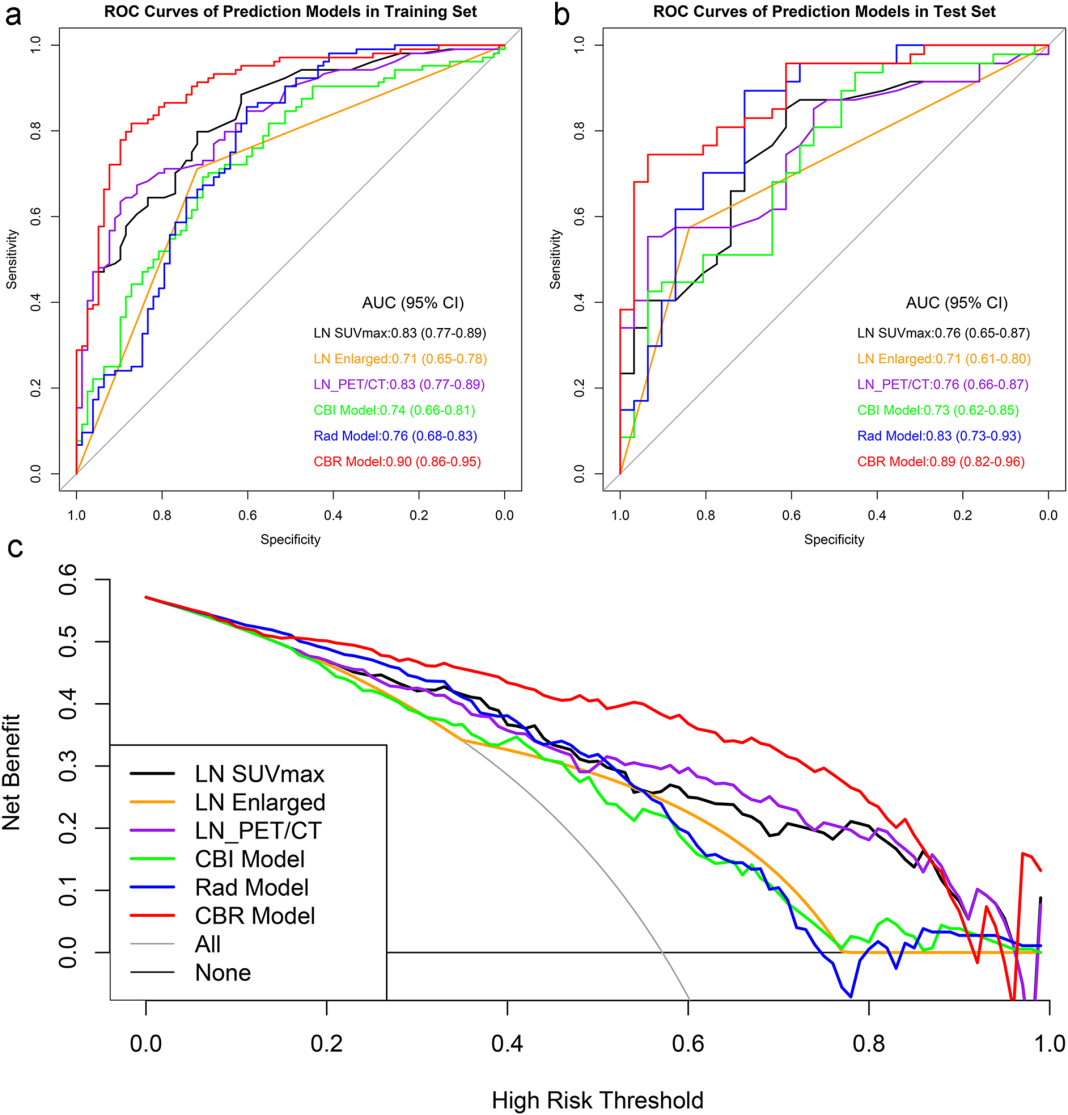
Receiver-operating characteristic analysis of models for predicting LNs status in the training set (**a**) and (**b**), respectively. Decision curve analysis of prediction models in the training set (**c**). The *X*-axis represented the threshold probability that was where the expected benefit of treatment was equal to the expected benefit of avoiding treatment. The *Y*-axis represented the net benefit. The grey and black line represented the hypothesis that all lung cancer patients were LN + and LN−, respectively

The performance of these 6 prediction models to discriminate LN− from LN + is shown in Fig. 4a, b. All the prediction models were significantly associated with the hypermetabolic mediastinal–hilar LN status, while the DeLong test showed that the CBR Model, which consisted of 1 clinical factor, 1 biological marker, 2 conventional PET/CT image features, 1 PET and 2 CT radiomics parameters, presented the lowest FPR and optimal discrimination among these models in both the training set (FPR of 12.82%, AUC of 0.90, and accuracy of 84.07%) and test set (FPR of 6.45%, AUC of 0.89, and accuracy of 82.05%) (both *p* < 0.05) (Table 3).

**Table 3:** Performance of models for predicting hypermetabolic mediastinal–hilar LNs status in lung cancer

Models	AUC (95% CI)	SEN	SPE	ACC	PPV	NPV	FPR	FNR
*Training set*								
LN SUVmax	0.83 (0.77–0.89)	79.81	71.79	76.37	79.05	72.73	28.21	20.19
LN enlarged	0.71 (0.65–0.78)	71.15	71.79	71.43	77.08	65.12	28.21	28.85
LN_PET/CT	0.83 (0.77–0.89)	67.31	85.90	75.27	86.42	66.34	14.10	32.69
CBI model	0.74 (0.66–0.81)	75.00	43.59	61.54	63.93	56.67	56.41	25.00
Rad model	0.76 (0.68–0.83)	85.58	60.26	74.73	74.17	75.81	39.74	14.42
CBR model	0.90 (0.86–0.95)	81.73	87.18	84.07	89.47	78.16	12.82	18.27
*Test set*								
LN SUVmax	0.76 (0.65–0.87)	85.11	61.29	75.64	76.92	73.08	38.71	14.89
LN enlarged	0.71 (0.61–0.80)	57.45	83.87	67.95	84.38	56.52	16.13	42.55
LN_PET/CT	0.76 (0.66–0.87)	55.32	93.55	70.51	92.86	58.00	6.45	44.68
CBI model	0.73 (0.62–0.85)	76.60	58.06	69.23	73.47	62.07	41.94	23.40
Rad model	0.83 (0.73–0.93)	89.36	70.97	82.05	82.35	81.48	29.03	10.64
CBR model	0.89 (0.82–0.96)	74.47	93.55	82.05	94.59	70.73	6.45	25.53

*AUC*  area under the receiver operating curve, *CI*  confidence interval, *SEN*  sensitivity, *SPE*  specificity, *ACC*  accuracy, *PPV*  positive predictive value, *NPV*  negative predictive value, *FPR*  false positive rate, *FNR*  false negative rate, *LN*  lymph node, *SUV*  standardized uptake value, *PET/CT*  positron emission tomography/computed tomography, *CBI*  clinico-biological-image, *Rad*  radiomics, *CBR*  clinico-biological-radiomics

Compared to the PET/CT, the CBR Model’s FPR decreased by 9.08%, while the AUC and accuracy separately increased by 8.43% and 11.69% in the training set. In the test set, the FPR of CBR Model was consistent with that of PET/CT, but its AUC and accuracy were significantly higher than PET/CT, with an increase of 17.11% and 16.37%, respectively.

The DCA also showed that the CBR Model was the most reliable clinical treatment tool for predicting the LN status in lung cancer when the threshold probability was greater than 18% (Fig. 4c).

### Individualized nomogram development and evaluation

**Fig. 5:**
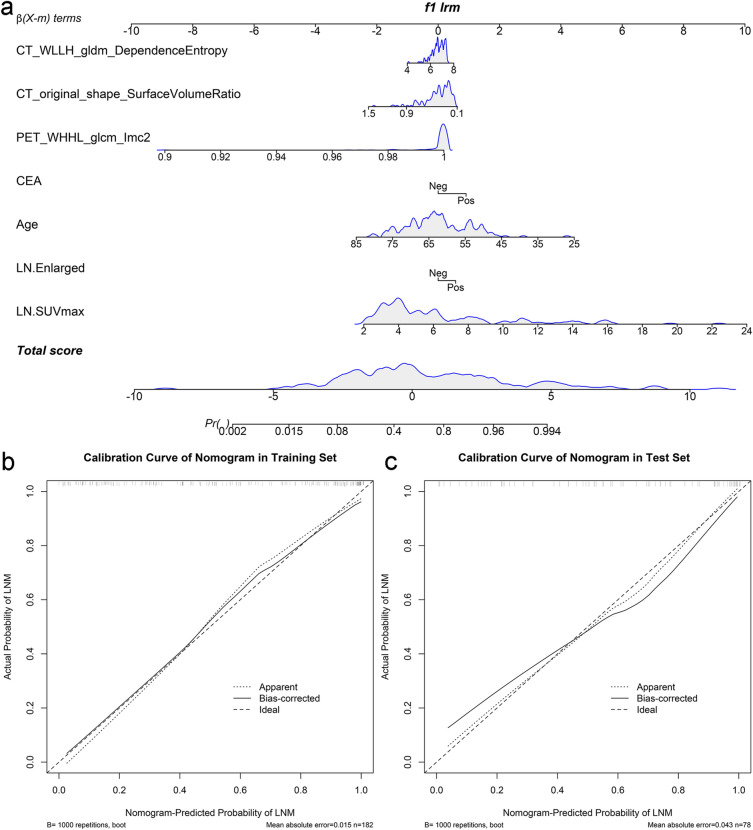
The nomogram was developed using the risk factors of CBR Model in the training set (**a**). The probability of each predictor could be converted into scores according to the first scale at the top of the nomogram. After adding up the corresponding prediction probability at the bottom of the nomogram was the risk of LNM. The nomogram’s score and probability threshold for predicting LNM were 0.19 and 0.55, respectively. Calibration curves showed the actual probability corresponded closely to the prediction of nomogram in training (**b**) and test (**c**) sets, respectively

**Fig. 6:**
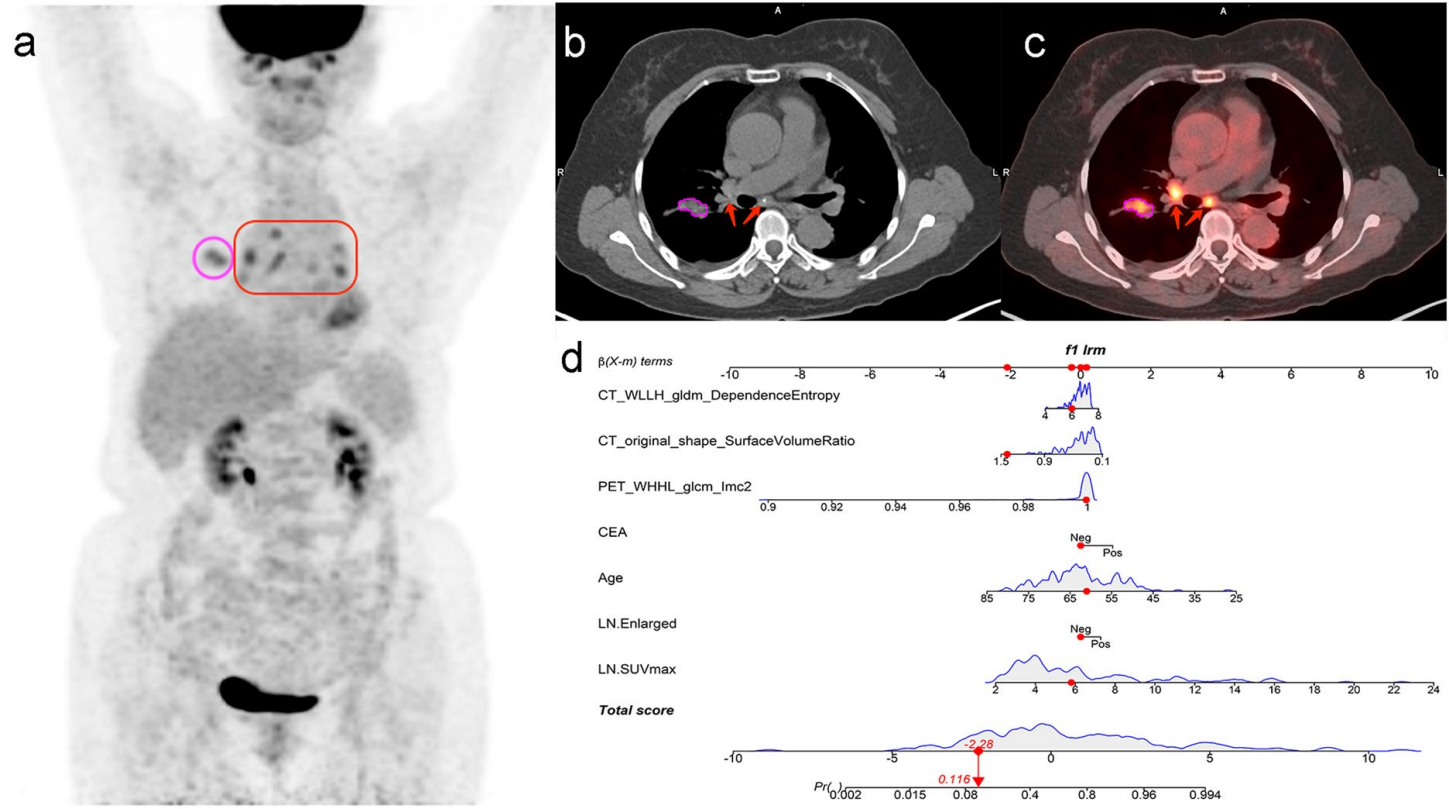
Example of nomogram clinical use. The preoperative whole-body PET/CT of this 61-year-old female with negative CEA status indicated the primary tumor was located in the right upper lobe (purple circle), with hypermetabolic mediastinal–hilar LNs (red square and arrows), and without distant metastasis (**a**–**c**). After completing the radiomics process (**b**, **c**) and applying the nomogram (**d**), the LNM probability of this patient was 0.12 (< 0.55), indicating a low risk of LNM. The pathological result of lobectomy with SND confirmed the negative status of mediastinal–hilar LN (0/7). The nomogram could improve the accuracy of hypermetabolic mediastinal–hilar LNs evaluation in lung cancer

According to the above results, an individualized nomogram based on the CBR Model’s risk factors was successfully developed for the visualization. The nomogram’s score and probability threshold for predicting LNM were 0.19 and 0.55, respectively (Fig. 5a). The calibration curves demonstrated a good agreement between the prediction of the LNM probability by the nomogram and the actual observation in both the training and test sets (Fig. 5b, c). Then, physicians could perform a pretherapeutic individualized prediction of the LNM risk to develop more reasonable and effective treatment plans for patients (Fig. 6).

## Discussion

In this study, we successfully explored a CBR nomogram incorporating multi-scale features, which held a more excellent performance in non-invasively N staging for lung cancer patients with hypermetabolic mediastinal–hilar LNs than conventional PET/CT, thereby greatly reducing the risk of overestimation and assisting for precision treatment.

Growing evidence suggests that radiomics integrated general CBI features achieve higher diagnostic efficacy than using them alone [30–32]. Thus, the clinico-biological factors of patients, PET/CT radiomics data of primary tumors, and image features of hypermetabolic mediastinal–hilar LNs were all applied to develop the prediction model in this study. Furthermore, on the basis of successfully screening features and establishing excellent models using a single ML algorithm (Lasso) in our previous study [17], we applied a combination of ML algorithms (mRMR + Lasso) to ensure the predictive performance of the model while minimizing the number of selected features to improve the model interpretability in the present study. The prediction performance of CBR Model with only 7 features established in this study was comparable to that of Combined Model with 14 features established in previous study. The result confirmed the feasibility of this approach.

Accurately identifying FP LNs is more challenging than assessing all LNs in lung cancer. To the best of our knowledge, only Ouyang et al*.* attempted a similar study using ML strategy [16]. They found that PET radiomics extracted from hypermetabolic mediastinal–hilar LNs integrated with CT image features could identify true and false positives of LNM in patients with non-small cell lung cancer with the highest AUC of 0.87. However, they mainly focused the role of LNs and did not concern the effect of the primary tumor. In this study, the CBR Model was developed using both the characteristics of the tumor and LNs, and validated to have more excellent potential in differentiating LN− (pN0) from LN + (pN1-2) patients in lung cancer (AUCs of 0.90 and 0.89 in the training and test sets, respectively). Moreover, the incorporating PET radiomics feature “WHHL_GLCM_Imc2” for characterizing tumor texture heterogeneity and CT radiomics feature “shape_SVR” for measuring tumor shape of CBR Model have also been selected in the Radiomics Model, indicating the robustness of these two features with high repeatability and reproducibility, which has also been confirmed in previous researches [33–35]. LN + patients generally had higher WHHL_GLCM_Imc2 and lower shape_SVR values than those in LN− patients (*p* < 0.05), suggesting that these two features were related to the tumor invasiveness, leading to a higher risk of LNM.

The accuracy of histologic staging of hypermetabolic LNs is also related to the clinico-biological-image factors. Patients’ age has been proven to be an independent risk factor, which means younger patients were more prone to having LNM, consistent with the positive status of pretherapeutic serum CEA [36]. Compared to conventional image tools, PET/CT is a significantly more accuracy non-invasive diagnostic procedure for LN staging in lung cancer, although it also has FP FDG-uptake in benign LNs [37]. Metastatic LNs generally have higher FDG uptake and bigger size than FP LNs (*p* < 0.05). However, it was difficult to achieve satisfactory prediction performance only using these conventional image parameters with the relatively higher AUC of 0.83. The efficiency of non-invasive LN prediction would increase by 8.43% in the case of CBR Model application. Simultaneously, the FPR of CBR Model for hypermetabolic mediastinal–hilar LNs evaluation was also outstanding with a decrease of 32.53 ~ 41.73% than previous clinical trials with the FPR of 19 ~ 22% [11, 12].

Furthermore, we generated an integrated nomogram on the basis of the CBR Model for facilitating its use in clinical practice. Then, the physicians could perform a preoperative individualized prediction of the LNM risk with this easy-to-use scoring tool, which could provide a non-invasive and accurate approach for patients who were unwilling or unable to undergo biopsy to develop more reasonable and effective treatment plans. The DCA also showed the nomogram added more benefit than either the treat-all-patients as LN− or the treat-all-patients as LN + , which was more valuable for the current trend toward personalized medicine [38, 39].

This study still has some limitations. Firstly, this retrospective study was conducted in a single center, which was the main cause of the decrease in RQS and also led to patient selection bias. It is necessary to design another prospective, multi-center, and large-cohort study to further validate the performance and generalization ability of the CBR Model in the real-world clinical settings [40]. Secondly, there is no significant statistical difference in primary tumor size, histologic type and metabolic parameters between LN− and LN + patients, consistent with the report [41]. This may be related to the weakened role of primary tumor in cases the included patients with both hypermetabolic tumor and LNs, equivalent to subgroup analysis. The sample size of patients, including ones with FDG-negative LNs, will be expanded to verify this hypothesis in further works. Thirdly, the radiomics analysis in this study only applied for primary tumor with semi-automatic segmentation, not LNs. This is due to the fact that larger tumors are more suitable for VOI segmentation that contribute to the robustness of study. The automatic segmentation approaches [42, 43] suitable for full volume VOI will be continually explored in future work.

In conclusion, an integrated CBR nomogram was successfully developed and validated in our study, which could further reduce the FPR and improve the accuracy of hypermetabolic mediastinal–hilar LNs evaluation in lung cancer than conventional PET/CT, thereby greatly reducing the risk of overestimation and assisting for precision treatment.

### Supplementary Information

**Additional file 1: Table S1. **Specific categories of radiomics features. **Table S2.** Complete clinicopathologic and metabolic factors of lung cancer patients.

## Data Availability

The datasets used and/or analyzed during the current study are available from the corresponding author on reasonable request.

## Abbreviations

AIC: Akaike’s information criterion
AUC: Area under curve
CA: Carbohydrate antigen
CBI: Clinico-biological-image
CBR: Clinico-biological-radiomics
CEA: Carcinoembryonic antigen
CI: Confidence interval
DCA: Decision curve analysis
FDG: Fluorodeoxyglucose
FNR: False negative rate
FP: False-positive
FPR: False positive rate
GLCM: Gray level co-occurrence matrix
GLDM: Gray level dependence matrix
GLRLM: Gray level run length matrix
GLSZM: Gray level size zone matrix
ICC: Intra- and inter-class correlation coefficient
Lasso: Least absolute shrinkage and selection operator
LN: Lymph node
LNM: Lymph node metastasis
ML: Machine learning
mRMR: Minimum-redundancy maximum-relevance
MTV: Metabolic tumor volume
NGTDM: Neighboring gray tone difference matrix
NPV: Negative predictive value
NSCLC: Non-small cell lung cancer
PET/CT: Positron emission tomography/computed tomography
PPV: Positive predictive value
Pre-score: Prediction score
Rad: Radiomics
ROC: Receiver-operating characteristic
RQS: Radiomics quality score
SD: Standard deviation
SND: Systematic lymph node dissection
SUV: Standardized uptake value
SVR: Surface volume ratio
TLG: Total lesion glycolysis
VOI: Volume of interest
