# Supplementary material for: Clinico-biological-radiomics (CBR) based machine learning for improving the diagnostic accuracy of FDG-PET false-positive lymph nodes in lung cancer
Source: Eur J Med Res. 2023 Dec 2;28:554. doi: 10.1186/s40001-023-01497-6 (PMC10693151; doi:10.1186/s40001-023-01497-6)
Supplement: Supplementary file 1 — Additional file 1: Table S1. Specific categories of radiomics features. Table S2. Complete clinicopathologic and metabolic factors of lung cancer patients. [file 40001_2023_1497_MOESM1_ESM.docx]

**Materials and methods**

***^18^F-FDG PET/CT image protocol and analysis***

All included patients in this study received the^18^F-FDG PET/CT examinations on the same equipment (Biograph 64 PET/CT scanner, Siemens Healthcare, Erlangen, Germany) with a blood glucose level of < 8.7 mmol/L fasted for at least 6 hours before the scan. After intravenous administration of 5.18 MBq/kg of ^18^F-FDG, the whole-body scan was operated about 1 hour later. First, CT scanning with 3.0 mm slice thickness (120 kVp, 150 mAs, 0.33 seconds per rotation) was performed and reconstructed to a 512 × 512 matrix (voxel size: 0.98 × 0.98 × 3.0 mm^3^). PET scanning was subsequently performed with 2 minutes in each bed and reconstructed using the TrueX algorithm (2 iterations, 24 subsets and 2 mm full width at half maximum, matrix size: 200 × 200, anisotropic voxels: 4.07 × 4.07 × 3.0 mm^3^). The PET images were transformed into SUV units by standardizing the activity concentration into the injected ^18^F-FDG’ dosage and patients’ weight.

**Table S1.** Specific categories of radiomics features

| Matrix | Radiomics Feature Name | Abbreviations |
| --- | --- | --- |
| Shape features (n=14) | Elongation | / |
|  | Flatness | / |
|  | Least Axis | / |
|  | Major Axis | / |
|  | Maximum 2D Diameter Column | M2DDC |
|  | Maximum 2D Diameter Row | M2DDR |
|  | Maximum2D Diameter Slice | M2DDS |
|  | Maximum3D Diameter | M3DD |
|  | Mesh Volume | / |
|  | Minor Axis | / |
|  | Sphericity | / |
|  | Surface Area | / |
|  | Surface Volume Ratio | SVR |
|  | Volume | / |
| First-order features (n=18) | 10 Percentile | / |
|  | 90 Percentile | / |
|  | Energy | / |
|  | Entropy | / |
|  | Interquartile Range | / |
|  | Kurtosis | / |
|  | Maximum | / |
|  | Mean Absolute Deviation | MAD |
|  | Mean | / |
|  | Median | / |
|  | Minimum | / |
|  | Range | / |
|  | Robust Mean Absolute Deviation | RMAD |
|  | Root Mean Squared | RMS |
|  | Skewness | / |
|  | Total Energy | / |
|  | Uniformity | / |
|  | Variance | / |
| Gray Level Co-occurrence Matrix (GLCM) (n=24) | Autocorrelation | / |
|  | Cluster Prominence | CP |
|  | Cluster Shade | CS |
|  | Cluster Tendency | CT |
|  | Contrast | / |
|  | Correlation | / |
|  | Difference Average | DA |
|  | Difference Entropy | DE |
|  | Difference Variance | DV |
|  | Id | / |
|  | Idm | / |
|  | Idmn | / |
|  | Idn | / |
|  | Imc1 | / |
|  | Imc2 | / |
|  | Inverse Variance | / |
|  | Joint Average | / |
|  | Joint Energy | / |
|  | Joint Entropy | / |
|  | MCC | / |
|  | Maximum Probability | / |
|  | Sum Average | / |
|  | Sum Entropy | / |
|  | Sum Squares | / |
| Gray Level Dependence Matrix (GLDM) (n=14) | Dependence Entropy | DE |
|  | Dependence Non-Uniformity | DNU |
|  | Dependence Non-Uniformity Normalized | DNUN |
|  | Dependence Variance | DV |
|  | Gray Level Non-Uniformity | GLNU |
|  | Gray Level Variance | GLV |
|  | High Gray Level Emphasis | HGLE |
|  | Large Dependence Emphasis | LDE |
|  | Large Dependence High Gray Level Emphasis | LDHGLE |
|  | Large Dependence Low Gray Level Emphasis | LDLGLE |
|  | Low Gray Level Emphasis | LGLE |
|  | Small Dependence Emphasis | SDE |
|  | Small Dependence High Gray Level Emphasis | SDHGLE |
|  | Small Dependence Low Gray Level Emphasis | SDLGLE |
| Gray Level Run Length Matrix (GLRLM) (n=16) | Gray Level Non-Uniformity | GLNU |
|  | Gray Level Non-Uniformity Normalized | GLNUN |
|  | Gray Level Variance | GLV |
|  | High Gray Level Run Emphasis | HGLRE |
|  | Long Run Emphasis | LRE |
|  | Long Run High Gray Level Emphasis | LRHGLE |
|  | Long Run Low Gray Level Emphasis | LRLGLE |
|  | Low Gray Level Run Emphasis | LGLRE |
|  | Run Entropy | RE |
|  | Run Length Non-Uniformity | RLNU |
|  | Run Length Non-Uniformity Normalized | RLNUN |
|  | Run Percentage | RP |
|  | Run Variance | RV |
|  | Short Run Emphasis | SRE |
|  | Short Run High Gray Level Emphasis | SRHGLE |
|  | Short Run Low Gray Level Emphasis | SRLGLE |
| Gray Level Size Zone Matrix (GLSZM) (n=16) | Gray Level Non-Uniformity | GLNU |
|  | Gray Level Non-Uniformity Normalized | GLNUN |
|  | Gray Level Variance | GLV |
|  | High Gray Level Zone Emphasis | HGLZE |
|  | Large Area Emphasis | LAE |
|  | Large Area High Gray Level Emphasis | LAHGLE |
|  | Large Area Low Gray Level Emphasis | LALGLE |
|  | Low Gray Level Zone Emphasis | LGLZE |
|  | Size Zone Non-Uniformity | SZNU |
|  | Size Zone Non-Uniformity Normalized | SZNUN |
|  | Small Area Emphasis | SAE |
|  | Small Area High Gray Level Emphasis | SAHGLE |
|  | Small Area Low Gray Level Emphasis | SALGLE |
|  | Zone Entropy | ZE |
|  | Zone Percentage | ZP |
|  | Zone Variance | ZV |
| Neighboring Gray Tone Difference Matrix (NGTDM) (n=5) | Busyness | / |
|  | Coarseness | / |
|  | Complexity | / |
|  | Contrast | / |
|  | Strength | / |
| Wavelet features (n=744) | LLH (n=93) | WLLH |
|  | LHL (n=93) | WLHL |
|  | LHH (n=93) | WLHH |
|  | HLL (n=93) | WHLL |
|  | HLH (n=93) | WHLH |
|  | HHL (n=93) | WHHL |
|  | HHH (n=93) | WHHH |
|  | LLL (n=93) | WLLL |

**Table S2.** Complete clinicopathologic and metabolic factors of lung cancer patients

| Factors | Total Set (n=260) | | *p* | Training Set (n=182) | | *p* | Validation Set (n=78) | | *p* |
| --- | --- | --- | --- | --- | --- | --- | --- | --- | --- |
|  | LN (-) (n=109) | LN (+) (n=151) |  | LN (-) (n=78) | LN (+) (n=104) |  | LN (-) (n=31) | LN (+) (n=47) |  |
| Gender, n (%) | | | 0.77 |  |  | 0.96 |  |  | 0.65 |
| Male | 85 (77.98) | 120 (79.47) |  | 62 (79.49) | 83 (79.81) |  | 23 (74.19) | 37 (78.72) |  |
| Female | 24 (22.02) | 31 (20.53) |  | 16 (20.51) | 21 (20.19) |  | 8 (25.81) | 10 (21.28) |  |
| Age (mean ± SD, years) | 64.94±6.98^†^ | 60.10±9.12^†^ | **< 0.01** | 65.10±7.19^†^ | 60.46±8.88^†^ | **< 0.01** | 64.52±6.53^†^ | 59.30±9.66^†^ | **0.01** |
| Height (m) | 1.65±0.08^†^ | 1.67±0.07^†^ | **0.02** | 1.66±0.08^†^ | 1.67±0.07^†^ | 0.08 | 1.65±0.08^†^ | 1.67±0.08^†^ | 0.16 |
| Weight (Kg) | 62.64±10.98^†^ | 66.81±10.30^†^ | **< 0.01** | 63.12±10.65^†^ | 66.92±10.17^†^ | **0.02** | 61.45±11.89^†^ | 66.55±10.69^†^ | 0.05 |
| BMI | 22.89±3.40^†^ | 23.79±3.02^†^ | **0.02** | 23.00±3.24^†^ | 23.81±2.97^†^ | 0.08 | 22.60±3.81^†^ | 23.75±3.17^†^ | 0.15 |
| Smoking, n (%) | | | 0.66 |  |  | 0.89 |  |  | 0.54 |
| Never | 36 (33.03) | 46 (30.46) |  | 24 (30.77) | 31 (29.81) |  | 12 (38.71) | 15 (31.91) |  |
| Ever/Always | 73 (66.97) | 105 (69.54) |  | 54 (69.23) | 73 (70.19) |  | 19 (61.29) | 32 (68.09) |  |
| Symptom, n (%) | | | 0.72 |  |  | 0.93 |  |  | 0.61 |
| Negative | 45 (41.28) | 59 (39.07) |  | 32 (41.03) | 42 (40.38) |  | 13 (41.94) | 17 (36.17) |  |
| Positive | 64 (58.72) | 92 (60.93) |  | 46 (58.97) | 62 (59.62) |  | 18 (58.06) | 30 (63.83) |  |
| Tumor History, n (%) | | | 0.31 |  |  | 0.35 |  |  | 0.61 |
| Negative | 92 (84.40) | 134 (88.74) |  | 67 (85.90) | 94 (90.38) |  | 25 (80.65) | 40 (85.11) |  |
| Positive | 17 (15.60) | 17 (11.26) |  | 11 (14.10) | 10 (9.62) |  | 6 (19.35) | 7 (14.89) |  |
| Tuberculosis History, n (%) | | | 0.24 |  |  | 0.23 |  |  | 0.67 |
| Negative | 103 (94.50) | 147 (97.35) |  | 74 (94.87) | 102 (98.08) |  | 29 (93.55) | 45 (95.74) |  |
| Positive | 6 (5.50) | 4 (2.65) |  | 4 (5.13) | 2 (1.92) |  | 2 (6.45) | 2 (4.26) |  |
| Family History, n (%) | | | 0.15 |  |  | 0.70 |  |  | 0.30 |
| Negative | 80 (73.39) | 98 (64.90) |  | 59 (75.64) | 76 (73.08) |  | 21 (67.74) | 22 (46.81) |  |
| Positive | 29 (26.61) | 53 (35.10) |  | 19 (24.36) | 28 (26.92) |  | 10 (32.26) | 25 (53.19) |  |
| Histological Type | | | 0.72 |  |  | 0.33 |  |  | 0.35 |
| ADC | 56 (51.37) | 89 (58.94) |  | 35 (44.87) | 61 (58.65) |  | 21 (67.74) | 28 (59.57) |  |
| SCC | 48 (44.04) | 48 (31.79) |  | 39 (50.00) | 33 (31.73) |  | 9 (29.03) | 15 (31.91) |  |
| Others | 5 (4.59) | 14 (9.27) |  | 4 (5.13) | 10 (9.62) |  | 1 (3.23) | 4 (8.52) |  |
| SCCA (ng/mL) | 1.15 (0.87, 1.97) ^‡^ | 1.06 (0.71, 1.70) ^‡^ | 0.52 | 1.12 (0.81, 1.91) ^‡^ | 1.00 (0.70, 1.70) ^‡^ | 0.70 | 1.39 (1.05, 1.97) ^‡^ | 1.48 (0.90, 1.60) ^‡^ | 0.43 |
| SCCA Status | | | 0.39 |  |  | 0.79 |  |  | 0.24 |
| Negative | 68 (62.39) | 102 (67.55) |  | 51 (65.38) | 70 (67.31) |  | 17 (54.84) | 32 (68.09) |  |
| Positive | 41 (37.61) | 49 (32.45) |  | 27 (34.62) | 34 (32.69) |  | 14 (45.16) | 15 (31.91) |  |
| CA199 (U/mL) | 11.64 (6.85, 18.60) ^‡^ | 13.35 (7.95, 22.81) ^‡^ | 0.11 | 11.07 (6.28, 18.62) ^‡^ | 12.45 (7.61, 23.48) ^‡^ | 0.08 | 15.97 (10.34, 18.60) ^‡^ | 17.40 (8.12, 22.27) ^‡^ | 0.68 |
| CA199 Status | | | 0.07 |  |  | 0.10 |  |  | 0.48 |
| Negative | 97 (88.99) | 122 (80.79) |  | 70 (89.74) | 84 (80.77) |  | 27 (87.10) | 38 (80.85) |  |
| Positive | 12 (11.01) | 29 (19.21) |  | 8 (10.26) | 20 (19.23) |  | 4 (12.90) | 9 (19.15) |  |
| CA125 (U/mL) | 13.57 (9.87, 20.99) ^‡^ | 17.04 (11.85, 26.63) ^‡^ | 0.17 | 12.65 (9.33, 21.07) ^‡^ | 15.68 (11.18, 24.85) ^‡^ | 0.40 | 17.02 (11.26, 20.99) ^‡^ | 19.02 (13.40, 28.51) ^‡^ | **0.04** |
| CA125 Status | | | 0.05 |  |  | 0.31 |  |  | **0.04** |
| Negative | 100 (91.74) | 126 (83.44) |  | 70 (89.74) | 88 (84.62) |  | 30 (96.77) | 38 (80.85) |  |
| Positive | 9 (8.26) | 25 (16.56) |  | 8 (10.26) | 16 (15.38) |  | 1(3.23) | 9 (19.15) |  |
| CA153 (U/mL) | 12.89 (8.55, 15.78) ^‡^ | 15.22 (10.52, 19.37) ^‡^ | **< 0.01** | 12.01 (7.85, 15.77) ^‡^ | 13.82 (10.38, 19.81) ^‡^ | **0.01** | 13.08 (12.02, 15.81) ^‡^ | 16.94 (11.62, 17.46) ^‡^ | 0.09 |
| CA153 Status | | | 0.14 |  |  | 0.06 |  |  | 0.67 |
| Negative | 102 (93.58) | 133 (88.08) |  | 73 (93.59) | 88 (84.62) |  | 29 (93.55) | 45 (95.74) |  |
| Positive | 7 (6.42) | 18 (11.92) |  | 5 (6.41) | 16 (15.38) |  | 2 (6.45) | 2 (4.26) |  |
| CEA (ng/mL) | 3.24 (2.07, 5.75) ^‡^ | 4.91 (2.75, 17.16) ^‡^ | **0.03** | 3.22 (2.03, 4.94) ^‡^ | 4.54 (2.73, 10.50) ^‡^ | 0.08 | 4.09 (2.20, 7.77) ^‡^ | 7.12 (2.94, 24.70) ^‡^ | 0.21 |
| CEA Status | | | **< 0.01** |  |  | **0.01** |  |  | 0.25 |
| Negative | 77 (70.64) | 80 (52.98) |  | 59 (75.64) | 59 (56.73) |  | 18 (58.06) | 21 (44.68) |  |
| Positive | 32 (29.36) | 71 (47.02) |  | 19 (24.36) | 45 (43.27) |  | 13 (41.94) | 26 (55.32) |  |
| CYFRA21-1 (ng/mL) | 3.77 (2.52, 5.94) ^‡^ | 4.51 (2.93, 7.07) ^‡^ | 0.98 | 3.66 (2.57, 5.63) ^‡^ | 4.33 (2.91, 7.39) ^‡^ | 0.86 | 3.86 (2.41, 6.85) ^‡^ | 5.53 (3.02, 6.96) ^‡^ | 0.06 |
| CYFRA21-1 Status | | | 0.12 |  |  | 0.22 |  |  | 0.33 |
| Negative | 48 (44.04) | 52 (34.44) |  | 34 (43.59) | 36 (34.62) |  | 14 (45.16) | 16 (34.04) |  |
| Positive | 61 (55.96) | 99 (65.56) |  | 44 (56.41) | 68 (65.38) |  | 17 (54.84) | 31 (65.96) |  |
| NSE (ng/mL) | 11.90 (10.13, 14.11) ^‡^ | 11.98 (10.40, 13.83) ^‡^ | 0.51 | 11.60 (9.87, 14.01) ^‡^ | 11.77 (10.21, 14.35) ^‡^ | 0.30 | 12.46 (10.71, 14.31) ^‡^ | 12.45 (10.46, 13.52) ^‡^ | 0.47 |
| NSE Status | | | 0.61 |  |  | 0.85 |  |  | 0.17 |
| Negative | 96 (88.07) | 136 (90.07) |  | 69 (88.46) | 91 (87.50) |  | 27 (87.10) | 45 (95.74) |  |
| Positive | 13 (11.93) | 15 (9.93) |  | 9 (11.54) | 13 (12.50) |  | 4 (12.90) | 2 (4.26) |  |
| Tumor Side, n (%) | | | 0.56 |  |  | 0.64 |  |  | 0.74 |
| Right Lung | 66 (60.55) | 86 (56.95) |  | 47 (60.26) | 59 (56.73) |  | 19 (61.29) | 27 (57.45) |  |
| Left Lung | 43 (39.45) | 65 (43.05) |  | 31 (39.74) | 45 (43.27) |  | 12 (38.71) | 20 (42.55) |  |
| Tumor Location, n (%) | | | 0.49 |  |  | 0.81 |  |  | 0.36 |
| Upper Lobe | 59 (54.13) | 90 (59.60) |  | 45 (57.69) | 62 (59.62) |  | 14 (45.16) | 28 (59.57) |  |
| Middle Lobe | 8 (7.34) | 7 (4.64) |  | 4 (5.13) | 5 (4.81) |  | 4 (12.90) | 2 (4.26) |  |
| Lower Lobe | 42 (38.53) | 54 (35.76) |  | 29 (37.18) | 37 (35.57) |  | 13 (41.94) | 17 (36.17) |  |
| Gross Type | | | **0.01** |  |  | 0.15 |  |  | **0.02** |
| Peripheral | 81 (74.31) | 90 (59.60) |  | 56 (71.79) | 64 (61.54) |  | 25 (80.65) | 26 (55.32) |  |
| Central | 28 (25.69) | 61 (40.40) |  | 22 (28.21) | 40 (38.46) |  | 6 (19.35) | 21 (44.68) |  |
| Tumor Size (cm) | 3.46±1.83^†^ | 4.04±1.89^†^ | **0.01** | 3.60±1.96^†^ | 4.10±1.81^†^ | 0.08 | 3.09±1.42^†^ | 3.91±2.04^†^ | 0.06 |
| Tumor SUVmax | 11.73±6.75^†^ | 11.79±5.70^†^ | 0.94 | 12.32±7.19^†^ | 12.33±6.03^†^ | 0.99 | 10.25±5.27^†^ | 10.60±4.72^†^ | 0.76 |
| Tumor SUVmin | 1.69±1.29^†^ | 1.48±0.92^†^ | 0.13 | 1.54±1.19^†^ | 1.52±0.84^†^ | 0.88 | 2.04±1.47^†^ | 1.38±1.08^†^ | **0.03** |
| Tumor SUVmean | 6.40±3.36^†^ | 6.29±2.78^†^ | 0.77 | 6.51±3.62^†^ | 6.58±2.91^†^ | 0.88 | 6.14±2.65^†^ | 5.65±2.36^†^ | 0.39 |
| Tumor MTV | 23.21±47.42^†^ | 30.09±35.42^†^ | 0.18 | 28.29±54.53^†^ | 30.57±35.20^†^ | 0.73 | 10.42±15.13^†^ | 29.01±36.25^†^ | **0.01** |
| Tumor TLG | 157.08±321.79^†^ | 205.25±276.95^†^ | 0.20 | 189.59±369.14^†^ | 215.89±273.04^†^ | 0.58 | 75.29±115.59^†^ | 181.69±286.97^†^ | 0.05 |
| LN Enlarged | | | **< 0.01** |  |  | **< 0.01** |  |  | **< 0.01** |
| Negative | 82 (75.23) | 50 (33.11) |  | 56 (71.79) | 30 (28.85) |  | 26 (83.87) | 20 (42.55) |  |
| Positive | 27 (24.77) | 101 (66.89) |  | 22 (28.21) | 74 (71.15) |  | 5 (16.13) | 27 (57.45) |  |
| LN SUVmax | 4.20±1.63^†^ | 7.65±4.07^†^ | **< 0.01** | 4.15±1.67^†^ | 7.86±4.11^†^ | **< 0.01** | 4.34±1.55^†^ | 7.19±3.96^†^ | **< 0.01** |

Note: †Values refer to mean ± standard deviation. ‡Values refer to median (interquartile range). *P* values were the results of univariate analysis and the bold ones indicated statistical significance.
